# Recycling of Pre-Washed Municipal Solid Waste Incinerator Fly Ash in the Manufacturing of Low Temperature Setting Geopolymer Materials

**DOI:** 10.3390/ma6083420

**Published:** 2013-08-12

**Authors:** Claudio Ferone, Francesco Colangelo, Francesco Messina, Luciano Santoro, Raffaele Cioffi

**Affiliations:** 1 INSTM Parthenope Research Unit, Department of Engineering, University of Naples Parthenope, Centro Direzionale Is. C4, Naples 80143, Italy; E-Mails: colangelo@uniparthenope.it (F.C.); francesco.messina@uniparthenope.it (F.M.); rcioffi@uniparthenope.it (R.C.); 2 Department of Chemical Sciences, University of Naples Federico II, Monte Sant’Angelo Complex, Naples 80126, Italy; E-Mail: luciano.santoro@unina.it

**Keywords:** stabilization, municipal solid waste incinerator fly ash, washing treatment, geopolymer bricks, coal fly ash

## Abstract

In this work, three samples of municipal solid waste incinerators fly ash (MSWI-FA) have been stabilized in systems containing coal fly ash to create geopolymers through a polycondensation reaction. Monolithic products have been obtained with both MSWI fly ash as received and after the partial removal of chloride and sulfate by water washing. The polycondensation products have been characterized qualitatively by means of Fourier transform infrared spectroscopy, X-ray diffraction and scanning electron microscopy and quantitatively, through the determination of the volume of reacted water and silicate. Furthermore, the heavy metals and chloride releases together with the physico-mechanical properties have been evaluated on the hardened products. In conclusion, considering the technological and environmental performances of the obtained geopolymers, they could be suitable for many non-structural applications, such as backfilling of abandoned quarries, decorative materials or brick fireplaces, hearths, patios, *etc.*

## 1. Introduction

The incineration of municipal solid wastes has relevant social, economic and environmental impacts. This process generates several gaseous effluents and produces both solid and liquid residues. The latter corresponds to 10% of initial waste volume. Hence, the proper management of these residues, particularly bottom and fly ash, is of crucial importance, and techniques for their stabilization/solidification need further optimization [1,2,3].

The aforementioned issue can find an answer in eco-design approaches which aim for material recovery to reduce the consumption of natural raw materials in the field of cement-based materials manufacturing. The unreactive stabilized waste can be employed together with solid wastes produced by other industrial processes. In fact, resource optimization implies significant advantages in terms of economic, energetic and environmental parameters of concrete industry (e.g., LEED—Leadership in Energy and Environmental Design Indicators). This industry is able to recycle and stabilize many kinds of solid wastes both in binder and artificial aggregates production, in order to achieve sustainability objectives [4,5,6,7,8,9,10,11,12].

Fly ash from municipal solid waste incinerators (MSWI-FA) are classified as hazardous in the European Union. Therefore, prior to a proper stabilization process, their contaminant release has to be evaluated. From this point of view, in addition to heavy metals, chlorides and sulfates pose major issues. In fact, untreated MSWI-FA release very high amounts of these pollutants when they are submitted to the leaching test UNI 10802-2004 [13] that derives from test EN 12457-2: 2002 [14]. These release values are always higher than the limits required for both hazardous and non-hazardous waste landfilling. Furthermore, any effective stabilization process is not economically sound if MSWI-FA are stabilized without a proper washing pretreatment. In this regard, attempts to optimize the solid/liquid ratio, with consequent water consumption reduction, can be found in the literature [15,16,17,18]. More specifically, Colangelo *et al.* [17] have applied one-step and two-step washing pretreatments on three different fly ash samples proving that the use of a very limited 2:1 overall liquid to solid ratio is possible. Thereby, the pre-washed MSWI-FA have been proposed as cement bound granular material in the manufacture of sub-base layer for road construction. Furthermore, a cost analysis of the complete process has been made too. Specifically, this cost analysis was carried out taking into account the charges for cement-stabilization, washing pretreatment and washing salt disposal less the benefits from material reuse and comparing this with the charge for untreated MSWI-FA simple disposal. The results have demonstrated that the proposed process is economically sound.

As far as the stabilizing matrices are considered, it is well known that cementitious ones based on cements, pozzolans, blast furnace slag and lime are often not suitable to reduce the very high mobility of chlorides and sulfates down to the imposed regulation limits. The reason for this is that high chloride and sulfate concentration has a strong negative effect on their efficiency [19,20]. Alternative matrices, such as those based on alkali-activated aluminosilicate binders, including the geopolymers, are worthy of consideration because excellent mechanical properties, durability, resistance to acid attack and thermal stability can be achieved. The synthesis of geopolymers takes place by polycondensation and can start from silicoaluminate and aluminosilicate materials. When they are in contact with the high pH of alkaline solution, raw materials dissolve and the inorganic polymers precipitate [21,22,23,24,25]. Recently, the applications of this broad class of materials in several fields of engineering have been deeply discussed by many authors, revealing a great number of possible technological solutions [26,27,28,29,30,31,32,33]. Great interest also derives from the possibility of employing naturally occurring silicoaluminate and aluminosilicate industrial wastes, such as coal fly ash, blast furnace slag, clay sediment, *etc.*, thus decreasing the environmental impact for the manufacturing of new materials based on geopolymers. In addition, the synthesis of neo-formed phases takes place at low temperatures, not higher than 60 °C [21,22,23,24,25,34,35,36,37]. All these considerations imply, in comparison to traditional cement-based materials, a reduction of natural raw materials consumption and greenhouse gases emission, particularly CO_2_.

In the field of hazardous solid waste treatment, the above polycondensation phases can favour the entrapment of contaminants, by means of both physical and chemical mechanisms, when geopolymers are employed as stabilizing matrices. Particularly, the stabilization/solidification of MSWI-FA in geopolymers has been already discussed by several authors in recent years [38,39,40,41,42,43]. Even if geopolymeric matrices setting and hardening are based on a different chemistry, as for the cementitious systems, the negative effect of the presence of chlorides and sulfates on the polycondensation kinetic was observed [44,45].

In order to optimize the entire cycle of MSWI-FA stabilization, water pre-washing can be applied for chlorides (and other soluble salts such as sulfates) removal. To this regards, Zheng *et al.* [46] investigated the effect of water-wash on geopolymerization. They concluded that a combined washing-stabilization process gave better immobilization efficiency of some heavy metals and higher early strength of hardened specimens. The drawbacks of this approach are the related water consumption for complete chlorides removal and the secondary pollution arising from the transfer of chlorides and other soluble salts from the ash to the washing water. So, the washing pretreatment must be optimized in relation to the minimum washing water requirement and maximum allowed residual amount of chlorides (and other soluble salts). Finally, an adequate binder to waste ratio is to be used in the stabilization process for the economically sound management of MSWI-FA landfilling/reuse.

In this work, coal fly ash has been used for the synthesis of geopolymeric matrices that can incorporate and stabilize three samples of fly ash from municipal solid wastes incinerators (MSWI). The different MSWI-FA samples have been used not only as received, but also after washing to reduce their chloride content. The products obtained under the different experimental conditions have been characterized from the qualitative point of view by means of Fourier transform infrared spectroscopy (FT-IR), X-ray diffraction (XRD) and scanning electron microscopy (SEM) and from the quantitative point of view through the measurement of the amounts of silicate and water reacted upon polycondensation. Finally, density and compressive strength of hardened specimens have also been evaluated and the environmental and technological classification of the final materials has been assessed by means of leaching tests and management considerations. The main goal of this work is gathering experimental data useful for the MSWI-FA management in relation to final reuse/utilization options. From this point of view, it is clear that, due to the intrinsic characteristics of the materials employed, hi-tech solutions will be precluded and options such as abandoned quarry filling or low temperature setting, soft brick manufacturing will be more appropriate.

## 2. Materials and Methods

The three MSWI-FA samples come from plants located in southern, central and northern Italy and have been named A, B and C, respectively. These samples have been collected downstream of the air pollution control (APC) device and comprised MSWI fly ash plus APC residue. The three samples have been submitted to chemical analysis, as described extensively in a previous work [17]. These three ash samples have been submitted to total acid digestion according to ASTM 5258-92 [47] and subsequent chemical analysis through inductively coupled plasma atomic emission spectrometry (ICP-AES) technique for the determination of metal contents. Chloride and sulfate content has been determined by means of the Mohr method and ionic liquid chromatography, respectively. All the measurements have been replicated nine times, and when reporting the data, mean values and standard deviations have been shown.

The three MSWI-FA have been characterized in terms of heavy metal, chloride and sulfate release by means of UNI 10802 [13] leaching tests. This is a test that makes use of deionized water with a liquid to solid ratio of 10.l/kg and, in case of granular wastes (size < 4 mm), has a duration of 24 h without leachant renewal. The release results have been previously reported together with the compulsory limits for landfilling of both hazardous and non-hazardous wastes (D.M. 27/09/2010) [48]. The authors reported that MSWI-FA must be stabilized in order to reduce the release of some heavy metals and, in addition, of chlorides. In fact, even if the final option is landfilling, the disposal is not allowed because sometimes both the hazardous and non-hazardous limits are exceeded. Specifically, the release of cadmium exceeds the two limits in the case of ash A and B. Chromium release exceeds the landfill disposal limit for non-hazardous wastes in the cases of all the ash, while lead release exceeds both the limits in the case of ash B and only the limit for non-hazardous wastes disposal in the cases of the other two ash. All of the three ash showed values of zinc release slightly higher than the two limits. In the case of chloride release, the values were always much higher than the two limits, while only in the case of ash B, the sulfate release slightly exceeded the limits.

Furthermore, the need for an optimized washing pretreatment of ash must also be explored to improve stabilization/solidification (S/S) process efficiency. In fact, to address an economically sound proposal, the waste amounts have to be maximized and, as a consequence, a preliminary washing treatment of ash could be worthy of consideration. Following the results reported by Colangelo *et al.* [17] in the above cited experimentation, a double step washing treatment with a water to ash ratio of 2:1 has been applied to the present work where a geopolymeric stabilizing system is studied. This kind of process, although more complex, minimizes water consumption, and therefore, contributing to the economy of the whole process. In fact, each ash sample has been divided into equal parts, and in the first step, one of them has been washed with a water to solid ratio of 4:1. During the second step, the solution coming from the first step is contacted with the other part of the ash. In this way, each part of ash is in contact with an amount of water useful for better handling of the liquid/solid suspension. Moreover, the overall liquid/solid ratio is 2:1.

Geopolymer systems produced from coal fly ash have been used as stabilizing matrices of the three different MSWI-FA. The coal fly ash used for the synthesis of the geopolymers has been supplied by the Italian electricity board (ENEL S.p.A., Rome, Italy) and comes from a power plant located in Brindisi (Southern Italy). It is the same as that used in a previous work [21] and its characterization, made by means of the same chemical analytical techniques as reported for MSWI-FAs, has given the following chemical composition: SiO_2_, 44.3% (2060 mg,_Si_/kg,_FA_) Al_2_O_3_, 20.2% (1070 mg/kg); Fe_2_O_3_, 10.5% (734 mg/kg); K_2_O, 8.1% (737 mg/kg); CaO, 0.5% (36 mg/kg); Na_2_O, 0.3% (22 mg/kg); loss on ignition at 1050 °C, 11.3%. Alkali activation, necessary to promote polycondensation, has been carried out by adding NaOH and sodium silicate solutions of proper concentration.

The three samples of MSWI-FA have been submitted to the stabilization treatment both as received, and after partial soluble salt removal (mainly chlorides and sulfates) carried out by the double step water washing previously described.

The compositions of the systems tested are reported in Table 2 and have been designed by fixing at 75/25 the MSWI-FA/coal fly ash ratio. Cylindrical samples (diameter 3 cm, height 6 cm) have been prepared by pouring each mixture into polyethylene moulds. Three samples have been cured for three days at 60 °C in oven under 100% relative humidity (RH) conditions. Afterwards, the specimens have been extracted from the moulds and subjected to Unconfined Compressive Strength (UCS) determination by using a 100 kN capacity Controls^®^ MCC8 testing machine.

This mechanical evaluation is significant because it is well known that rapid strength development is a peculiar feature of geopolymerization.

It is important to underline that in previous works the MSWI-FA/solid precursor ratios were much lower than 75/25. Specifically, Lancellotti *et al.* [40] employed systems based on metakaolin/MSWI-FA mixtures with about 17% ash, while Luna Galiano *et al.* [41] used about 26% ash in respect to coal fly ash in geopolymeric systems based on coal fly ash/MSWI-FA, coal fly ash + blast furnace slag/MSWI-FA, coal fly ash + metakaolin/MSWI-FA and coal fly ash + kaolin/MSWI-FA.

The complete set of experimental compositions is reported in Table 1. These compositions have been designed taking into account those studied in the previous work [21] that gave good geopolymerization results and also by considering that a large portion of coal FA is replaced by MSWI-FA in this work. The components of all the systems listed in Table 2 have been carefully mixed and the resulting mixtures have been kept in small polyethylene cylinders of size *d* × *h* = 3 cm × 6 cm. The polycondensation reaction has been carried out at 25 °C for times equal to 1, 3, 7, 14 and 28 days.

**Table 1:** Composition of the geopolymer materials, wt %.

System	MSWI-FA	Coal fly ash	Sodium silicate solution (1.15 M)	NaOH solution
GA_AR_^1^	48	16	18	18 (10 M)
GA_W_^2^	51.5	16.5	16	16 (10 M)
GB_AR_	57.5	18.5	12	12 (10 M)
GB_W_	60	20	10.5	10.5 (10 M)
GC_AR_	53	17	15	15 (17 M)
GC_W_	55	18	13.5	13.5 (17 M)

Notes: ^1^ GX_AR_: geopolymer mixture containing MSWI-FA type X (X = A or B or C) as received; ^2^ GX_w_: geopolymer mixture containing MSWI-FA type X (X = A or B or C) pre-washed.

The specimens obtained at any prefixed polycondensation time have been characterized by means of a Thermo Scientific Nicolet Nexus FT-IR spectrometer (Thermo Scientific, Waltham, MA, USA) equipped with a DTGS KBr (deuterated triglycine sulfate with potassium bromide windows) detector. FT-IR absorption spectra have been recorded in the 4000–400 cm^−1^ range. A spectral resolution of 2 cm^−1^ has been chosen. 2.0 mg of each test sample has been mixed with 200 mg of KBr in an agate mortar, and then pressed into 200 mg pellets of 13 mm diameter. The spectrum of each sample represents an average of 32 scans. Furthermore, a Philips PW 1730 X-ray diffractometer (Philips, Eindhoven, The Netherlands) (CuKα radiation, 40 kV, 40 mA, 2θ range from 10° to 80°, equivalent step size 0.0179° 2θ, equivalent counting time 120 s per step) has been employed in order to obtain the mineralogical characterization of the same series of samples. Selected hardened samples have been also submitted to a microstructural characterization by means of a FEI Quanta 200 FEG scanning electron microscope (FEI, Hillsboro, OR, USA).

The same specimens have been used for the quantitative determination of water and sodium silicate consumed during the polycondensation reaction. The amounts of reacted sodium silicate and water at any polycondensation time have been determined as follows. Each specimen has been ground under acetone, filtered and washed with diethyl ether to remove all the residual aqueous phase. Finally, the samples have been heated in an oven up to 40 °C in order to ensure the loss of any residual fraction of the liquids previously used. The cumulative amount of reacted sodium silicate and water has been obtained by weight difference between the solid recovered after the above treatments and the ash initially employed. The amount of reacted water has been determined by the excess loss on ignition of the recovered solid over that of the initial ash. This method is extensively described in previous works [21,35].

The leaching behaviour of the stabilized systems has been assessed submitting cubic specimens of 4 cm in size to UNI 10802 test (UNI 10802, 2004) [13]. This procedure follows the protocol for monolithic specimens, which imposes water renewals after 2 and 18 h, for a total duration of 48 h. The solid surface to liquid ratio has been fixed at 1:10. At the end of each test, the pH of leachate has been measured.

To evaluate the suitability of the stabilized/solidified geopolymeric systems containing pre-washed ash for material reuse, three series of cubic specimens of 4 cm in size have been cured for 28 days at room temperature and 100% RH. Then, they have been submitted to density evaluation and UCS measurements making use of the same testing machine described above.

## 3. Results and Discussion

### 3.1. MSWI-FA Chemical Characterization and Geopolymerization

The results of the chemical analysis are shown in Table 2. In this work, an evaluation of the most abundant oxides was also made. The reported results revealed amounts of CaO, SiO_2_ and Al_2_O_3_ in the ranges 23.0–32.2, 15.2–20.4 and 5.2–10.2 wt %, respectively. These data have been taken into account for the formulation of the stabilizing geopolymeric systems proposed in the present work.

**Figure 1:**
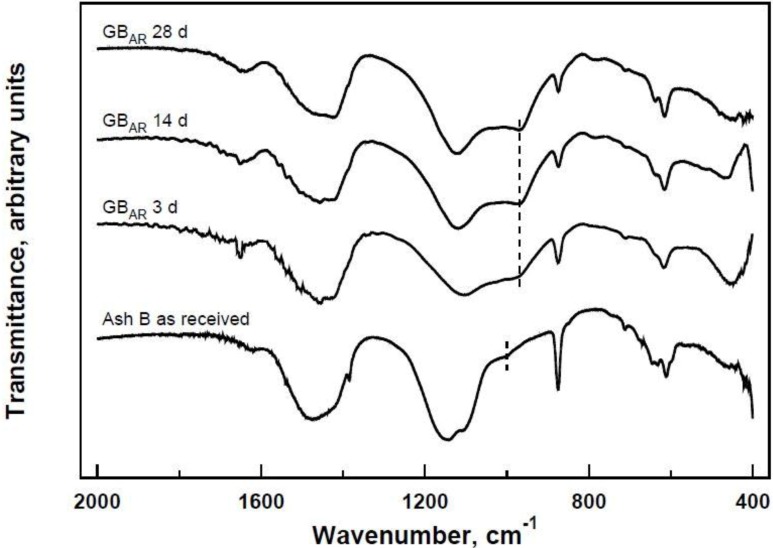
FT-IR characterization of system GB_AR_ at several selected polycondensation times.

Infrared spectroscopy is a useful tool for revealing the formation of geopolymers. In fact, in FT-IR traces of raw silicates and silico-aluminates, the Si–O asymmetric stretching in tetrahedra is responsible for an absorption band centred at about 1000 cm^−1^. When geopolymers are formed, this band is shifted to lower wavenumbers as a consequence of polycondensation with alternating Si–O and Al–O bonds (see dashed lines in Figure 1). This phenomenon can be clearly seen in Figure 1, where the results of FT-IR characterization are reported for system GB_AR_ at some selected polycondensation times.

**Table 2:** Chemical composition of municipal solid waste incinerators (MSWI) fly ash, mg/kg.

Component	Samples								
	A			B			C		
Ca	230,000	±	11,200	270,000	±	11,700	165,000	±	9,900
Cl^−^	113,000	±	8,100	49,000	±	3,700	75,000	±	4,800
Si	110,000	±	1,800	130,000	±	2,200	97,000	±	2,100
SO_4_^2−^	29,000	±	5,500	68,000	±	11,500	34,000	±	7,200
Na	15,000	±	1,050	28,000	±	1,530	119,000	±	9,800
Fe	12,000	±	2,300	10,700	±	2,040	9,450	±	1,790
Al	12,000	±	380	27,000	±	1,050	14,000	±	440
K	11,300	±	1,080	17,000	±	1,140	24,000	±	1,840
Zn	9,100	±	530	6,230	±	430	8,400	±	440
Pb	8,950	±	460	17,110	±	980	6,580	±	270
Mg	8,500	±	210	7,500	±	190	1,240	±	40
Cu	815	±	59	6,220	±	390	4,114	±	220
Ni	130	±	6	163	±	8	117	±	6
Ba	112	±	22	227	±	43	185	±	37
Cr_tot_	85	±	24	270	±	75	412	±	95
Cd	65	±	13	217	±	41	88	±	15
As	4.2	±	1.3	5.9	±	1.7	2.1	±	0.9

**Figure 2:**
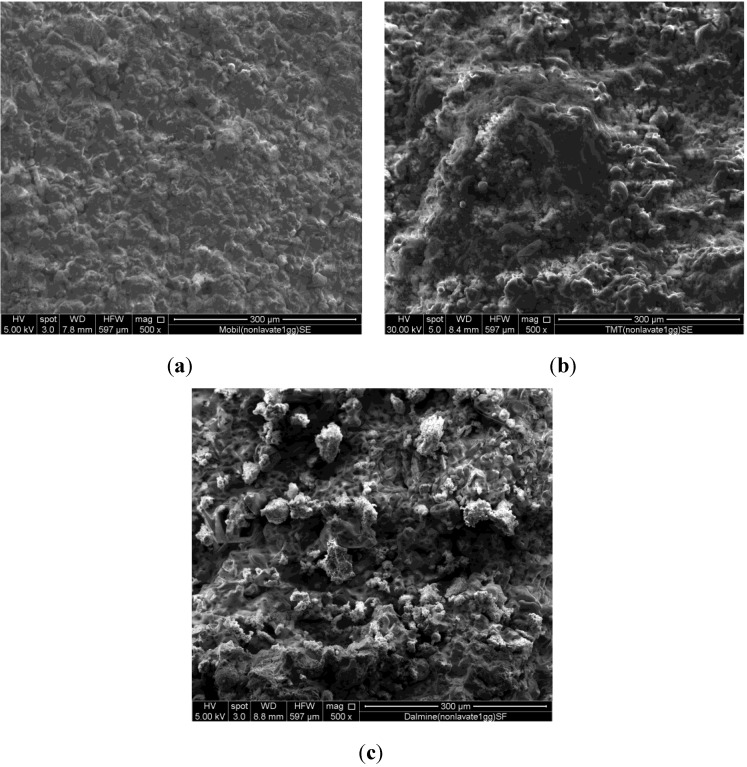
SEM Micrographs of systems (**a**) GA_AR_ 28 days; (**b**) GB_AR_ 28 days and (**c**) GC_AR_ 28 days.

The band originally present at 1032 cm^−1^ in the trace relative to ash B as received shifts to 977 cm^−1^ after a polycondensation time of 28 days. In addition, the intensity of this band increases with time, indicating a corresponding increase of polycondensation degree. Figure 2 shows the micrographs of the three systems investigated after 28 days of curing. The three systems contain the ash A(a), B(b) and C(c) as received (*i.e*., without partial soluble salts removal).

**Figure 3:**
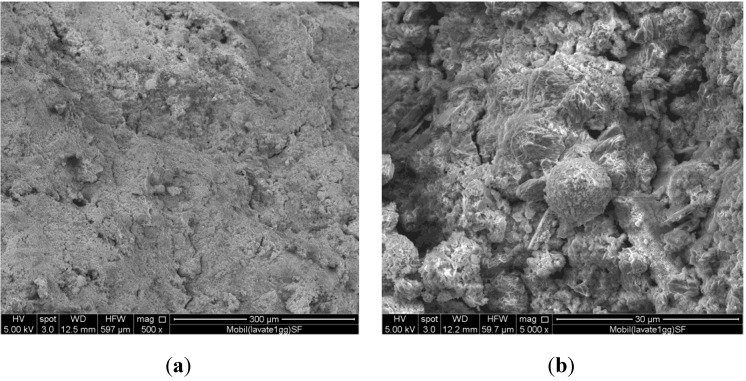
SEM Micrographs of system containing washed A ash, (**a**) 500× and (**b**) 5000× magnifications.

Despite the fact that the results of FT-IR investigation show that polycondensation takes place in all the systems, the morphology of the cured samples containing the ash as received does not appear so compact to favour the development of good physico-mechanical properties. This observation holds for all the ash, even if the content of soluble salts is quite different from case to case. Figure 3 shows the micrographs of the system containing A ash previously washed and cured for 28 days (the same time considered for the unwashed systems of Figure 2).

**Figure 4:**
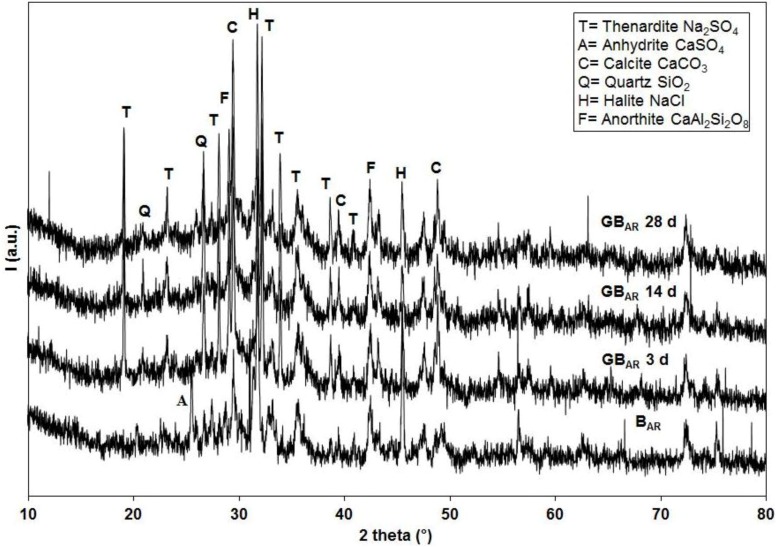
X-ray diffraction (XRD) patterns of B_AR_, GB_AR_ 3 days, GB_AR_ 14 days and GB_AR_ 28 days samples.

In this case, the specimens morphology looks more compact (Figure 3a), able to favour higher strength values. Furthermore, Figure 3b shows that polycondensation actually takes place; in fact, amorphous N-A-S-H gel-phase produced during the reaction grow on the reactive coal fly ash particles. Figure 4 shows the X-Ray diffraction patterns of sample B_AR_ and of samples GB_AR_ after 3, 7 and 28 days of curing.

All patterns show several crystallographic peaks in a substantially amorphous matrix. The major crystalline phases identified in the sample B_AR_ are Halite (NaCl, JCPDS card No. 5-628), Calcite (CaCO_3_, JCPDS card No. 5-586), Anhydrite (CaSO_4_, JCPDS card No. 37-1496), together with a low amount of Quartz (SiO_2_, JCPDS card No. 46-1045) and Anorthite (CaAl_2_Si_2_O_8_, JCPDS card No. 41-1486). Mineralogical composition of the fly ash is in good agreement with literature data [49]. Geopolymerized GB_AR_ samples substantially contain the same crystalline phases as the B_AR_ sample. Noteworthy is the disappearance of the Anhydrite and the appearance of Thenardite (Na_2_SO_4_, JCPDS card No. 37-1465), almost certainly due to the addition of a high amount of Na in the geopolymerization process. The distinguishing feature of the diffractogram of any geopolymer is a broad “hump” centered at approximately 27°–29° 2θ [50]. In Figure 4, a slight increase of this hump can be observed in the geopolymerized samples in respect to the as-received MSWI-FA. The same considerations can be made analysing the XRD patterns of the other two MSWI-FA samples employed.

The effect of pre-washing on the crystalline phase content of MSWI-FA is very similar to that observed by other authors [46]. Water washing determines the disappearance or decrease in intensity of the chlorides and sulfates containing phases. The XRD patterns (data not shown) of geopolymeric specimens containing pre-washed MSWI-FA do not show significant differences in terms of the above cited amorphous hump aspect.

The quantitative data of reacted water and silicate are reported in Table 3 and Table 4 for all the systems studied and at all the polycondensation times investigated. The data of Table 3 show that the amount of water bound to the geopolymers decreases as the polycondensation time increases. This is a direct consequence of the reaction mechanism: initially, the starting materials dissolve in the highly alkaline reaction medium giving rise to the formation of geopolymer precursors in which several hydroxyl groups are present; then, crosslinking of these precursors takes place and the polycondensation occurs with water expulsion [51].

**Table 3:** Amount of reacted water in mg/g of initial ash.

System	Polycondensation time (days)				
	1	3	7	14	28
**GA_AR_**	65.6	51.3	51.7	49.9	37.9
**GA_W_**	83.8	59.7	46.6	43.5	36.5
**GB_AR_**	72.3	41.3	51.7	9.9	0.7
**GB_W_**	59.8	59.7	46.6	32.5	24.5
**GC_AR_**	17.9	41.3	21.3	19.9	12.3
**GC_W_**	108.4	63.7	54.6	62.5	64.5

**Table 4:** Amount of reacted silicate in mg/g of initial ash.

System	Polycondensation time (days)				
	1	3	7	14	28
**GA_AR_**	91.8	109.2	105.2	111.9	120.8
**GA_W_**	92.3	98.8	104.8	112.8	113.9
**GB_AR_**	47.4	68.2	70.2	78.9	100.8
**GB_W_**	69.3	69.8	74.8	82.8	82.9
**GC_AR_**	47.4	69.2	30.2	78.9	60.8
**GC_W_**	89.3	130.4	100.7	102.8	65.9

The data of Table 4 show that, despite a few exceptions, the amount of reacted silicate increases with reaction time. The quantitative data of Table 4 can be compared with similar results obtained in the previous work [21] in which coal FA was used on its own. In the above cited work, it was found that starting with SiO_2_/Al_2_O_3_ ratios equal to 4 and 6, the amount of reacted silicate at 25 °C and after 28 days, reached the values of about 200 and 250 g/g of initial FA, respectively. In this work, the presence of MSWI-FA worsens these results, but not dramatically, inasmuch as values ranging from about 60–120 mg/g are reached under the same experimental conditions (see Table 4). Some differences can be seen in relation to MSWI-FA origin, but in all the cases, the degree of polycondensation is high enough to get monolithic products. Reducing the content of chlorides by washing has a minor effect, if any. This is particularly relevant in relation to MSWI-FA stabilization, as it is well known that the effectiveness of traditional cement-based matrices can be severely compromised by high chloride content.

### 3.2. MSWI-FA Stabilization for Safer Disposal

The results of the leaching tests carried out on the stabilized specimens containing MSWI-FA as-received are reported in Table 5. If these results are compared with those reported by Colangelo *et al.* in a previous work [17], it can be seen that the geopolymer-based stabilizing system is more efficient in respect to a cement-based one. The values reported in parentheses are relative to systems where an 80/20 MSWI-FA to cement ratio has been imposed and the ash have been mixed without a washing pretreatment. The authors found that the process had a limited positive effect on the leaching behavior of chlorides and sulfates.

**Table 5:** Results of UNI 10802 leaching test on stabilized systems containing MSWI-FA as-received, mg/L.

Components	System			Limits for non-hazardous wastes
	GA_AR_	GB_AR_	GC_AR_	
As	<0.10 (0.10)	<0.10 (0.12)	<0.10 (<0.10)	0.2
Ba	0.17 (0.34)	<0.10 (<0.1)	0.31 (0.57)	10
Cd	<0.10 (0.18)	<0.10 (0.33)	<0.10 (<0.10)	0.1
Cr_tot_	0.91 (1.31)	0.67 (0.91)	0.45 (0.98)	1
Ni	0.18 (0.53)	0.21 (0.98)	0.14 (0.75)	1
Pb	0.52 (1.31)	1.14 (1.52)	0.50 (0.91)	1
Cu	0.10 (0.15)	1.18 (4.18)	0.43 (1.01)	5
Zn	1.64 (1.69)	1.12 (1.82)	0.87 (0.97)	5
Cl^−^	5080 (7140)	2115 (3015)	3450 (4780)	1500
SO_4_^2−^	1080 (1480)	3160 (4150)	1570 (1830)	2000

Note: Results of previous cement-stabilization/solidification process with MSWI-FA/cement = 80/20.

The data of Table 5 show that, although the geopolymer system is more effective in respect to pollutant release, the resulting values for chlorides are still higher than the limits imposed by Italian regulation (D.M. 27/09/2010, 2010) [48] for disposal of stabilized wastes in landfill for non-hazardous wastes. The improvement of effectiveness is partially relevant for chlorides, as the release is reduced by 29%, 30% and 28% in the case of systems GA_AR_, GB_AR_ and GC_AR_, respectively. Despite the better immobilization compared to what was found by Colangelo *et al.* [17], release of sulfates and lead even exceeds the above limits for system GB_AR_. If these results are compared with those obtained by other authors on geopolymer-based stabilizing systems, including MSWI-FA, it is possible to see that the most important findings are in agreement. Lancellotti *et al.* [40] studied the stabilization of two MSWI-FA samples by employing a metakaolin-based geopolymer matrix. In the cited work, the wastes were stabilized without a specific washing pretreatment with a metakaolin/MSWI-FA ratio of 5/1. After curing, the leaching behavior and the chemical stability of the matrix were assessed showing that the systems could be disposed of in a landfill for non-hazardous wastes. Luna Galiano *et al.* [41] used systems containing coal fly ash, blast furnace slag, metakaolin and kaolin in different ratios to stabilize an unwashed MSWI-FA sample. A comparison with ordinary Portland cement and lime-based systems was made through compressive strength and leaching behavior evaluation. Zheng *et al.* [43] employed coal fly ash-based geopolymer binders to evaluate both the effect of Si/Al ratio and alkali content on heavy metal release and the microstructure of systems containing untreated MSWI-FA. Particularly, Lancellotti *et al.* [40], in agreement with others researches [38,39,41,42,43], found that cadmium is highly immobilized in geopolymer matrices due to the very low solubility of Cd(OH)_2_ in the highly alkaline leachate of the coal fly ash-based geopolymer system. In addition, the leaching behavior of nickel, chromium, copper and lead is also comparable.

As in this case, Luna Galiano *et al.* [41] reported comparisons between the stabilizing efficiency of various coal fly ash/geopolymer and cementitious mixtures. They found similar differences in leaching behavior between the two different binding systems. As far as the chloride release is concerned, the values detected in our systems prove that a pre-washed treatment of fly ash is required. Table 6 shows the release values of the stabilized geopolymer-based systems containing the MSWI-FA after the two-step washing treatment.

**Table 6:** Results of UNI 10802 leaching test on stabilized systems containing two-step 2:1 washed MSWI-FA, mg/L.

Components	System			Limits for non-hazardous wastes
	GA_w_	GB_w_	GC_w_	
As	<0.10 (0.10)	<0.10 (0.12)	<0.10 (<0.10)	0.2
Ba	0.11 (0.31)	<0.10 (<0.10)	0.28 (0.38)	10
Cd	<0.10 (0.17)	<0.10 (0.23)	<0.10 (<0.10)	0.1
Cr_tot_	0.80 (1.10)	0.53 (0.80)	0.43 (0.53)	1
Ni	<0.10 (0.50)	0.16 (0.73)	<0.10 (0.58)	1
Pb	0.47 (1.47)	1.05 (1.34)	0.31 (0.81)	1
Cu	0.10 (0.17)	0.71 (3.71)	0.53 (0.93)	5
Zn	1.61 (1.71)	1.07 (1.57)	0.81 (0.91)	5
Cl^−^	1240 (1840)	1410 (910)	1160 (1170)	1500
SO_4_^2−^	480 (630)	550 (450)	580 (460)	2000

Note: Results of previous cement-stabilization/solidification process with MSWI-FA/cement = 80/20.

It can be seen that, as in the previous study of the same fly ash [17], the chloride extraction strongly reduces the release values measured according to the UNI 10802 [13] leaching test. The measured values are lower than the limits fixed for disposal of stabilized (unreactive) wastes. As expected, all the heavy metal release values decrease below the limits and consequently, the stabilized systems are suited for safer disposal if landfilling is the final disposal option.

The economic advantages of this final option are evident considering the possibility to dispose of the MSWI-FA in a less expensive landfill for non-hazardous wastes. It is so because the overall water requirement is limited, even if the two-step pre-treatment seems to be more complex.

In all the leaching steps, the pH has been measured and the detected values were always highly alkaline (>11). This is strongly associated to the nature of the mixtures and agrees with previous findings on similar systems where different MSWI-FA have been mixed in various ratios with coal fly ash, metakaolin, kaolin and blast furnace slag [38,39,40,41,42,43].

### 3.3. MSWI-FA Stabilization/Solidification and Management for Material Reuse

**Figure 5:**
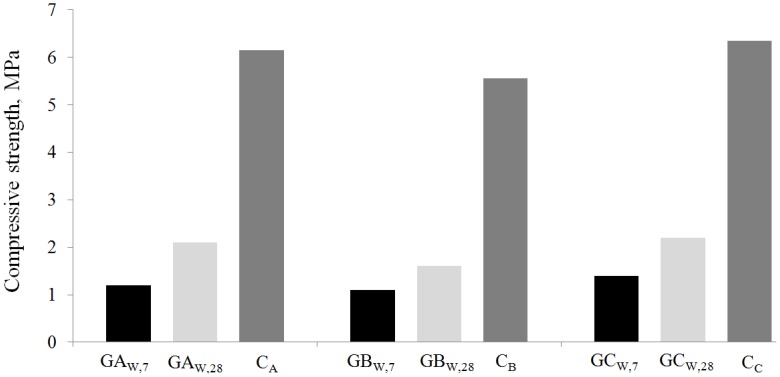
Compressive strength of MSWI-FA geopolymeric cubic specimens.

The results of UCS measurements on the three hardened geopolymeric systems, after 7 and 28-day curing, are shown in Figure 5 together with the values previously measured on cement-based solidified systems containing MSWI-FA cured for 28 days and proposed as bound granular material for road basement [17].

In consideration of the high fire-resistance and hardness of geopolymeric materials, the experiments were carried out on hardened specimens in order to achieve a final product in the field of decorative or non-structural applications, such as brick fireplaces, hearths, patios, *etc.*

The manufacturing of geopolymer bricks (geobricks) based on alkali activated coal fly ash-based systems was already studied by Palomo *et al.* [52]. They explored possible applications of geobricks as monoblock, lightweight matrices and fire resistant tiles where geopolymer typical technological properties can be advantageously exploited.

In other studies, Ariöz *et al.* [53,54] produced geobricks making use of coal fly ash, sodium hydroxide and sodium silicate solution curing the mixtures at up to 75 °C and in the presence of forming pressure. Furthermore, a number of commercial manufacturing processes have already been developed showing interesting market areas for the final products [55,56]. In all the cited cases, the role of both curing temperature and forming pressure were underlined in terms of strength, density, porosity, heat conductivity, *etc.* The results showed that also at low curing temperature, the systems investigated gave a technical performance adequate for a wide range of applications. It can be seen that the data of geopolymer type systems cured at room temperature are lower than that of cementitious ones. In particular, the mechanical performances of all the type G mixtures are very similar with the only exception for the system containing type B MSWI-FA. In fact, the higher chlorides residual content present in ash B gives, on the corresponding system, a slightly lower compressive strength than that shown by the other two mixtures. This is in agreement with the findings of the microstructural characterization. The values of the density measured on specimens cured for 28 days are: 1420, 1340 and 1435 kg/m^3^ for the systems GAw, GBw and GCw, respectively. Also, in this case, a comparison with cement-based systems is carried out. The trend of the results agrees with that observed in Figure 5.

All the measured absolute physico-mechanical values are quite close to that of soft masonry stones like clay or zeolite-based Neapolitan yellow tuff bricks. The mean compressive strength of the latter type of stones ranges between 2 and 5.73 MPa, while the density values are in the range of 1500–1650 kg/m^3^, as reported in a recent wide investigation on compressive behavior of tuff masonry panels [57]. The entire set of physical and mechanical data shows that, in line with this view, the stabilization with 25% of binder is technologically sound for the proposed way of material reuse.

In Italy, specific authorization is needed before material reuse can be put into practice by means of hazardous waste stabilization processes. The MSWI-FA stabilization process studied in this work can only be considered an economically interesting proposal and a feasible technique for material reuse in the field of backfilling in abandoned quarries. To this regard, due to the specific geology of many areas present in Campania Region and the consequent huge amounts of quarried tuff stones, it can easily be evaluated that the proposed application could absorb very high quantities of stabilized MSWI-FA.

As far as the process economy is concerned, considerations similar to those presented in the previous paper, where cement stabilization was proposed, can be made. In that case, the complete ash treatment process cost (together with the washing-salt disposal) was estimated to be cheaper than the non-hazardous landfill disposal [17]. In the case of geopolymer stabilization process, this alternative treatment of MSWI-FA could be considered less expensive and more environmentally friendly. In fact, even if the cost of geopolymer matrix components is not yet standardized, the possibility to employ industrial solid waste, such as coal fly ash, is a very attractive option.

## 4. Conclusions

This work has proved that mixtures containing coal fly ash and pre-washed MSWI-FA can be employed for the synthesis of geopolymeric systems. Three different samples of MSWI-FA have been used and in all the cases the polycondensation took place with formation of monolithic products. The experiments have been also carried out with MSWI-FA in which the soluble salt content had been significantly lowered by a water washing process optimized in relation to water consumption.

Leaching tests have been carried out on both as-received and washed MSWI-FA, showing that the geopolymer-based matrix has a better stabilizing effect in comparison to previously studied cementitious systems. Chemical and microscopic analyses proved that the content of soluble salts plays a minor role in the amounts of reacted water and silicate, but strongly affects the microstructure of the neo-formed phases.

Finally, it can be argued that, considering the results of the physico-mechanical tests, MSWI-FA washing could be very advantageous from the point of view of safer ash disposal and their recycling as backfilling blocks for abandoned quarries or low temperature setting geopolymer soft bricks.
